# Elevating Insulin Signaling Using a Constitutively Active Insulin Receptor Increases Glucose Metabolism and Expression of GLUT3 in Hippocampal Neurons

**DOI:** 10.3389/fnins.2020.00668

**Published:** 2020-07-07

**Authors:** Hilaree N. Frazier, Adam O. Ghoweri, Katie L. Anderson, Ruei-Lung Lin, Gabriel J. Popa, Michael D. Mendenhall, Lawrence P. Reagan, Rolf J. Craven, Olivier Thibault

**Affiliations:** ^1^Department of Pharmacology and Nutritional Sciences, University of Kentucky College of Medicine, Lexington, KY, United States; ^2^Department of Molecular and Cellular Biochemistry, University of Kentucky College of Medicine, Lexington, KY, United States; ^3^Department of Pharmacology, Physiology and Neuroscience, University of South Carolina School of Medicine, Columbia, SC, United States

**Keywords:** hippocampus, neuron, astrocyte, glucose metabolism, GLUT3, signaling

## Abstract

Insulin signaling is an integral component of healthy brain function, with evidence of positive insulin-mediated alterations in synaptic integrity, cerebral blood flow, inflammation, and memory. However, the specific pathways targeted by this peptide remain unclear. Previously, our lab used a molecular approach to characterize the impact of insulin signaling on voltage-gated calcium channels and has also shown that acute insulin administration reduces calcium-induced calcium release in hippocampal neurons. Here, we explore the relationship between insulin receptor signaling and glucose metabolism using similar methods. Mixed, primary hippocampal cultures were infected with either a control lentivirus or one containing a constitutively active human insulin receptor (IRβ). 2-NBDG imaging was used to obtain indirect measures of glucose uptake and utilization. Other outcome measures include Western immunoblots of GLUT3 and GLUT4 on total membrane and cytosolic subcellular fractions. Glucose imaging data indicate that neurons expressing IRβ show significant elevations in uptake and rates of utilization compared to controls. As expected, astrocytes did not respond to the IRβ treatment. Quantification of Western immunoblots show that IRβ is associated with significant elevations in GLUT3 expression, particularly in the total membrane subcellular fraction, but did not alter GLUT4 expression in either fraction. Our work suggests that insulin plays a significant role in mediating neuronal glucose metabolism, potentially through an upregulation in the expression of GLUT3. This provides further evidence for a potential therapeutic mechanism underlying the beneficial impact of intranasal insulin in the clinic.

## Introduction

The brain, once thought to possess no circulating insulin or functional insulin receptors (IRs), has now been identified as an insulin-sensitive and insulin-dependent organ. Indeed, insulin signaling is not only associated with normal, healthy brain function and development (Shanker and Pieringer, 1988; Unger et al., 1991; Sena and Ferret-Sena, 1995; Schechter et al., 1998; Schulingkamp et al., 2000; Banks et al., 2012), but is also directly involved with important cognitive processes such as memory and learning (Craft et al., 1996; Biessels et al., 1998; Zhao et al., 1999; Zhao and Alkon, 2001; Adzovic et al., 2015). Additionally, a reduction in insulin binding, signaling, and receptor density, particularly in the hippocampus, has been associated with aging, Alzheimer’s disease (AD), and mild cognitive impairment (Tchilian et al., 1990; Zaia and Piantanelli, 1996; Dore et al., 1997; Frolich et al., 1998; reviewed in Hoyer, 1998; Messier and Teutenberg, 2005; reviewed in Cholerton et al., 2011; Biessels and Reagan, 2015). Therapeutic approaches aimed at offsetting these changes by increasing the amount of available insulin in the brain have recently been developed with great success. Of these, administration of intranasal insulin (INI) appears to be the most promising, as it provides a relatively safe, non-invasive, and effective method for bypassing the blood-brain barrier and delivering the ligand directly to the brain (reviewed in Schwartz et al., 1990; Israel et al., 1993; Baura et al., 1996; Craft, 2005; Banks et al., 2012; Heni et al., 2014; Chapman et al., 2018). While clinical studies have reported positive impacts of INI on learning and memory (Kern et al., 1999; Benedict et al., 2004; Reger et al., 2006; Craft et al., 2012; reviewed in de la Monte, 2013), it is still unclear whether the canonical IR signaling pathway mediates these effects given that several potential mechanisms have been suggested. These include the impact of insulin on cerebral blood flow (Akintola et al., 2017; Kullmann et al., 2017), its ability to attenuate markers of neuroinflammation (Beirami et al., 2017; Rajasekar et al., 2017), oxidative stress (reviewed in Matioli and Nitrini, 2015; Mamik et al., 2016), and age- or AD-related calcium dysregulation (Chik et al., 1997; Pancani et al., 2013; reviewed in Thibault et al., 2013; Maimaiti et al., 2016, 2017), and the presence of studies highlighting insulin-mediated improvements of neuronal glucose metabolism (Uemura and Greenlee, 2006; McNay et al., 2010; Pancani et al., 2011; Liu et al., 2015; Brabazon et al., 2017; Chen et al., 2018).

In the periphery, canonical IR signaling triggers activation of the phosphoinositide 3-kinase (PI3K) pathway, which in turn promotes translocation of glucose transporter (GLUT) 4 to the plasma membrane and facilitates glucose uptake into muscle and adipose tissue (reviewed in Klip et al., 2019). While the brain and periphery express two distinct isoforms of the IR (IR-A and IR-B, respectively), the overall structures of these receptors are generally comparable. Indeed, while IR-A in the brain has a higher affinity for insulin (Mosthaf et al., 1990) and is internalized at a much slower rate than the peripheral IR-B (reviewed in Joost, 1995), evidence shows that both receptors signal through the PI3K pathway and activate many of the same downstream effectors; thus, it is not unreasonable to assume that IR signaling may also induce GLUT4 activity in the brain. In fact, although the primary neuronal GLUT is GLUT3 (Bell et al., 1990; Vannucci et al., 1997), recent studies have reported the presence of the insulin-sensitive GLUT4 in both the cerebellum and the hippocampus (reviewed in Rayner et al., 1994; Kobayashi et al., 1996; Vannucci et al., 1998; Choeiri et al., 2002). Further, in the hippocampus, which also possesses high levels of IR (Unger et al., 1989; Kar et al., 1993; Dore et al., 1997), several labs have shown that insulin increases GLUT4 translocation and uptake of glucose, subsequently improving hippocampal processes (Piroli et al., 2007; Grillo et al., 2009; Pearson-Leary and McNay, 2016; Pearson-Leary et al., 2018). These same groups have also reported that administration of intracerebroventricular (ICV) insulin in rats increased GLUT4 translocation to the plasma membrane of hippocampal neurons in a PI3K-dependant manner (Grillo et al., 2009). Similar work in several cell lines align with these results, showing that acute *in vitro* insulin administration can induce translocation of GLUT4 to the plasma membrane (Benomar et al., 2006; Varshney and Dey, 2016). Interestingly, however, another recent study using a neuron-specific GLUT4 knock-out mouse model reported no change in hippocampal uptake of the glucose analog 2-deoxyglucose (2-DG) in these animals compared to wild-type controls (Reno et al., 2017). The limited body of work on GLUT3 in hippocampal cells suggests that other potential metabolic targets of central insulin signaling should be further investigated. In light of this, we chose to explore the relationship between insulin signaling, glucose kinetics, and the expression of GLUT3, as well as GLUT4, in hippocampal neurons and astrocytes.

Prior studies of insulin actions on hippocampal metabolism have primarily focused on acute exposures of the ligand to neurons (∼30 min). However, as insulin has been shown to influence downstream signaling targets such as glycogen synthase kinase 3 β (GSK3β), mitochondrial membrane potentials, and ATP production for sustained periods of time (∼24–30 h) (Huang et al., 2005), we chose to investigate the impact of chronic, long-term (96 h) IR activation on glucose handling in mixed, primary hippocampal cell cultures. Although cell culture models are not fully representative of *in vivo* brain metabolism, the chronic, continuous administration of high insulin to the brains of animals may not be preferable given the potential impact on peripheral glucose (Stockhorst et al., 2004; Gelling et al., 2006). Therefore, use of an *in vitro* approach allows us to directly investigate the effects of long-term IR signaling on neurons and astrocytes while minimizing additional confounding variables that could be present *in vivo* (i.e., changes in CBF, satiety status, and other peripheral hormones). Additionally, while low nanomolar concentrations of insulin are often employed to selectively bind the IR, the possibility for non-specific activation of other known or unknown receptors (i.e., IGF-I) still remains. For this reason, we opted to induce elevated insulin signaling by expressing a genetically modified human IR using a previously validated molecular approach (Frazier et al., 2018). This receptor, IRβ, is comprised almost solely of the intracellular catalytic β subunit, yet is still capable of translocating and successfully inserting into the plasma membrane (Lebwohl et al., 1991). The truncation of the α subunit renders the modified receptor constitutively active, as highlighted by our previous work showing that IRβ significantly elevates pAKT levels for at least 48 h in the same cell culture model used here (Frazier et al., 2018). Thus, this technique allows us to reliably increase IR signaling in the absence of exogenous insulin.

Here, we show that long-term, sustained IR signaling conferred by the constitutively active IRβ receptor significantly increased uptake and indirect measures of utilization of the glucose analog 2-[N-(7-nitrobenz-2-oxa-1,3-diazol-4-yl)amino]-2-deoxy-glucose (2-NBDG) in cultured hippocampal neurons, but not astrocytes. These results were corroborated using radiolabeled glucose assays, which also indicated elevated uptake in IRβ-treated dishes compared to controls. Additionally, we also report an IRβ-associated increase in the overall expression of GLUT3 in these cells, particularly in the total membrane subcellular fraction. Surprisingly, no differences in GLUT4 expression were detected. Our results further support the hypothesis that insulin signaling is tied to neuronal glucose metabolism in the hippocampus, possibly through the neuron-specific GLUT3. Additionally, these findings provide insight into potential mechanisms mediating the therapeutic benefits of INI in the clinic and highlight the validity of using molecular techniques to study these effects.

## Materials and Methods

### Preparation of Mixed, Primary Hippocampal Cell Cultures

Mixed (neurons and glia), primary hippocampal cell cultures were established from Sprague Dawley rat pups at embryonic day 18 or 19 as described previously (Porter et al., 1997; Pancani et al., 2009, 2011). Briefly, hippocampi were first dissected in ice-cold Hank’s balanced salt solution (Thermo Fisher Scientific, Waltham, MA, United States) supplemented with 4.2 mM NaHCO_3_ and 12 mM HEPES, then transferred to a 50 mL conical tube containing 0.25% trypsin-EDTA and incubated at room temperature (23°C) for 11 min. Hippocampi were subsequently washed three times with warm (37°C) SMEM [supplemented (200 mM L-glutamine and 35 mM D-glucose) Minimum Essential Medium (Thermo Fisher Scientific)], then triturated in 10 mL of warm SMEM. Cells were diluted to the desired concentration, plated onto coated (0.5% poly-L-lysine) 35 mm plastic (Corning Inc., Corning, NY, United States) or glass (Matsunami Glass Ind., Ltd., Osaka, Japan) dishes in 2 mL aliquots, and incubated at 37°C, 5% CO_2_. Plating densities (∼400,000 cells per dish) were recorded and later used to normalize tritiated (^3^H)-glucose uptake values for each experiment. Three days after plating, half of the media in each dish was replaced with 1 mL of a 5-fluoro-2-doxyuridine (FUDR) solution to stop glial cell growth. Because previous work in culture has demonstrated that glucose oxidation rates and insulin sensitivity in glial cells may be sensitive to the high concentrations of glucose in the growth medium (Abe et al., 2006), we purposefully returned cultures to a serum-free, normal glucose (5.5 mM) solution for 24 h prior to all experiments. Previous work from our lab has shown that this protocol maintains cellular viability, as evidenced by normal (∼100 nM) resting calcium measures (see Pancani et al., 2011). All data presented here were obtained at room temperature between days *in vitro* (DIV) 14–17.

### Lentiviral Construction and Delivery

Using a lentiviral delivery system, mixed, primary hippocampal cultures received one of two plasmids: a control plasmid containing a neuron-specific synapsin promoter and a fluorescent marker (mCherry), or an experimental plasmid containing the synapsin promoter, mCherry, and the constitutively active IRβ receptor. Both plasmids were constructed from a pHR-SFFV-KRABdCas9-P2A-Cherry backbone vector (gift from Jonathan Weissman, plasmid #60954, Addgene, Watertown, MA, United States) as described previously (Frazier et al., 2018). Briefly, the synapsin promoter and IRβ sequence were ligated between the AscI and BamHI sites using PCR and standard digestion protocols. The plasmids were converted into lentiviruses by co-transfecting HEK293 cells with the donor plasmid, PsPAX2, and pMD2.G (gifts from Dr. Didier Trono, plasmid #12260 and #12259, Addgene). The viruses were then precipitated into a pellet using 1.4% w/v polyethylene glycol and 50 mM NaCl, resuspended in cold PBS, and frozen (−80°C) until needed.

All dishes were infected on DIV 10 at a multiplicity of infection (MOI) of 25. Dishes were then immediately returned to the incubator for 48 h to allow ample time for protein expression. Routine confirmation of mCherry fluorescence was performed on both control and IRβ dishes using a spectral analysis camera (Nuance, CRi Inc., Boston, MA, United States). The expression rate of mCherry was ∼80% of cells per dish, similar to that reported in our prior IRβ study in this same cell culture model (Frazier et al., 2018). As with our previous study (Frazier et al., 2018), plasmid expression appeared to be limited to only neurons, as no detectable fluorescence was noted in astrocytes.

### 2-NBDG Imaging of Hippocampal Neurons and Astrocytes

To encourage uptake of 2-NBDG, hippocampal cultures were incubated in 3 mL of a HEPES-based imaging solution (10 mM HEPES, 145 mM NaCl, 2.5 mM KCl, 2 mM CaCl_2_, 1 mM MgCl_2;_ pH 7.3) that contained 0 mM D-glucose for 15 min at room temperature and air. This short protocol is necessary to prevent uptake competition between glucose and 2-NBDG (Pancani et al., 2011). Following the initial 15 min incubation, dishes received 200 μM 2-NBDG (diluted in sterile ddH_2_0 and added directly to each dish) and were then incubated for an additional 5 min in darkness. While longer exposures to 2-NBDG (2–4 h) result in increased uptake of the glucose analog, differences in the rate of fluorescence decay between neurons and astrocytes still remain (Pancani et al., 2011). Following 2-NBDG treatment, dishes were washed three times in supplemented (10 mM D-glucose) imaging solution, incubated in 3 mL of this same solution, and placed on the microscope stage (E600FN; Nikon Inc., Melville, NY, United States) at room temp and air for 3 min. During this 3 min time period, a field of view (FOV) containing morphologically distinct neurons and astrocytes that positively expressed mCherry (based on red fluorescence) was randomly selected (1 FOV per dish) using a 40x immersion objective. 2-NBDG imaging (exciter centered at 475 ± 40 nm, emitter centered at 535 ± 45 nm, dichroic mirror with a high-pass at ∼505 nm; no binning) began immediately after this final 3 min incubation. Sequential images (500 ms exposure) were taken every 30 s for 5 min for a total of 10 images. Phase images of each FOV were also captured and later used to ensure that only morphologically distinct, healthy neurons and astrocytes were included in the analysis.

**FIGURE 1:**
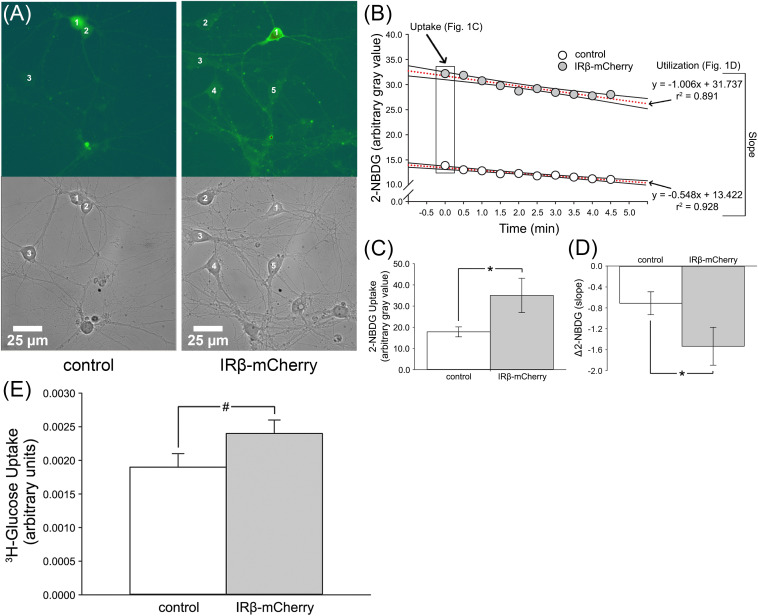
2-NBDG imaging and relative levels of ^3^H-glucose uptake in mixed, primary hippocampal neurons with or without expression of IRβ. **(A)** Representative phase and 2-NBDG fluorescent photomicrographs obtained from hippocampal cultures. Numbers 1–5 indicate distinct neurons. **(B)** Data obtained from a representative control and IRβ-expressing neuron during 2-NBDG imaging. Boxed data at time point 0.0 indicate initial gray values used for 2-NBDG uptake analysis. Dashed red lines indicate linear regressions used for Δ2-NBDG calculation (slope). **(C)** Quantification of background-subtracted 2-NBDG uptake in hippocampal neurons with or without IRβ expression. Significant elevation in 2-NBDG uptake was observed in IRβ-expressing neurons (*n* = 50 dishes) compared to controls (*n* = 42 dishes) (Student’s *t*-test, *p* = 0.046). **(D)** Quantification of background-subtracted change in 2-NBDG fluorescence (Δ2-NBDG) used to infer indirect rates of glucose utilization. A significant increase in the rate of 2-NBDG utilization was observed in IRβ-expressing neurons (*n* = 25) compared to controls (*n* = 21) (Student’s *t*-test, *p* = 0.044). **(E)**^3^H-glucose uptake measures were derived from a total of 3 independent cultures (*n* = 3 dams, 21 dishes per group). A strong trend for an elevation in radiolabeled glucose uptake was observed in the IRβ-expressing dishes compared to controls (Student’s *t*-test, *p* = 0.120). All data represent means ± SEM. Asterisks (*) indicate significance at *p* < 0.05. Hash (#) indicates trend at *p* < 0.15.

Fluorescent levels (arbitrary gray value) were quantified using Imaging Workbench 5.0 (Indec BioSystems, Santa Clara, CA, United States). Using each FOV’s phase image as a reference, a region of interest (ROI) was placed around the somatic area of the neurons and astrocytes. Distinction between the two cell-types was determined using cell morphology. For each dish, an additional ROI was also placed in an area devoid of any cellular components in order to obtain background signal, which was then subtracted from the 2-NBDG fluorescence values. As previously reported (Pancani et al., 2011), 2-NBDG uptake measures were derived from the initial image obtained (Figure 1B, boxed region), and rates of 2-NBDG utilization were determined by calculating the fluorescent signal decay over time (Figure 1B, slope). We report 2-NBDG imaging results from a total of 89 dishes (226 cells, 12 dams). Data are presented as means ± standard error of the mean (SEM).

### Radiolabeled Glucose Uptake

Glucose uptake assays using ^3^H-glucose were performed on control and IRβ-treated cultures between DIV14–15. Cells were first washed with PBS, then incubated in 1 mL PBS containing 0.1 mM 2-DG and 1 mCi/mL ^3^H-2-DG (Perkin Elmer, Boston, MA, United States) at 37°C for 5 min. Cells were then washed with ice-cold PBS and subsequently solubilized in 0.4 mL of 1% SDS for 10 min at room temperature. Cells were counted in 4 mL of Biosafe II Complete Counting Cocktail (Research Products International, Mount Prospect, IL, United States) for 1 min using a Beckman LS6500 scintillation counter (Beckman-Coulter Inc., Brea, CA, United States). To control for the potential impact of IRβ expression on cell numbers, raw scintillation values from each dish were normalized to the dish’s optical density [obtained using a bicinchoninic acid assay (BCA) protein quantification kit (Thermo Fisher Scientific)]. However, due to a lack of sample volume in one experiment, this normalization could only be performed on 2 of the 3 experiments. To control for variations in cell density across individual experiments (n’s), uptake values were then further normalized to the plating density obtained during initial cell culture preparation. Normalized ^3^H-glucose uptake values from all dishes were then combined and averaged within their treatment group. We present measures derived from a total of 42 dishes (21 control and 21 IRβ) across 3 individual experiments (*n* = 3 dams). Data are reported as group means ± SEM.

### Subcellular Fractionation and Western Immunoblots

For Western immunoblots, total membrane, cytosolic, and plasma membrane fractions were isolated using a modified subcellular fractionation protocol (Reagan et al., 2000; Piroli et al., 2002). Briefly, 8–16 dishes per treatment group were washed with 600 μL of room temperature PBS, lifted in 400–500 μL of a HEPES-based homogenizing buffer (320 mM sucrose, 2 mM EDTA, 2 mM EGTA, 20 mM HEPES) containing protease and phosphatase inhibitors (#P8340 and #P5726, respectively; Sigma-Aldrich, St. Louis, MO, United States), transferred to a sterile 2 mL microcentrifuge tube, homogenized using a Dounce homogenizing pestle (30 strokes), and spun in an ultracentrifuge at 800 × *g*, 4°C, for 10 min. The supernatant was removed and transferred to a fresh 1.5 mL tube while the remaining pellet was then resuspended in 100 μL of homogenizing buffer and spun again (800 × *g*, 4°C, 10 min). This second supernatant was then added to the first supernatant tube. A portion (∼250 μL) of the combined supernatant described above was aliquoted into a separate sterile tube and labeled as the “total membrane fraction.” The rest of the supernatant was then spun at 16,000 × *g*, 4 °C, for 30 min. The supernatant from this final spin was removed, placed in a new sterile tube, and labeled as the “cytosolic fraction.” The remaining pellet was resuspended in 250 μL of ice-cold RIPA-based buffer (25 mM Tris HCl, 150 mM NaCl, 1 mM EDTA, 1% NP-40, 1% sodium deoxycholate, 0.1% SDS, pH 7.6) containing protease and phosphatase inhibitors and labeled the “plasma membrane fraction.” Protein levels were determined using a BCA protein quantification kit and microplate reader. Samples were frozen at −20°C until used. To assess the efficacy of the fractionation, Western immunoblots for either whole-cell or membrane-only protein markers (GAPDH and calnexin, respectively) were performed on fractionated hippocampal cultures. As expected, GAPDH (known to be expressed throughout the cell) showed a clear signal all fractions tested (Supplementary Figure S1A), while calnexin, a protein often associated with neuronal endoplasmic reticulum and plasma membranes (Goebel et al., 2005; Itakura et al., 2013), was only detected in the total and plasma membrane fractions (Supplementary Figure S1B). This indicates that the cytosolic fraction was successfully separated from the two membrane fractions.

Immunoblots for GLUT3 and GLUT4 were performed only on total membrane and cytosolic fractions from hippocampal cultures (derived from *n* = 4 experiments across 5 dams, 8–16 dishes per group) in either duplicate or triplicate within and across gels. Plasma membrane pellets did not undergo GLUT3/4 immunoblotting, as this fraction yielded substantially less protein compared to the others (0.1–0.2 μg/mL vs. 1.0–1.5 μg/mL, respectively). Total protein load was semi-quantified using Ponceau S staining. To ensure that this method of total protein quantification is reliable, a comparison of Ponceau S (performed before probing) and Coomassie staining (performed after probing) was conducted on the same membrane from a subset of samples. Results showed similar uniformity across individual lanes in addition to similar relationships between virus treatments and subcellular fractions irrespective of the stain used (Supplementary Figure S2); thus, it does not appear that the staining method impacted our results.

Following Ponceau-S staining, target proteins were assessed using the following: 1° antibodies – GLUT3 #ab41525 1:1000 (Abcam, Cambridge, United Kingdom), and GLUT4 #SC18 1:1000 (gift from Dr. Lawrence Reagan, University of South Carolina); 2° antibody – anti-rabbit HRP-linked IgG #7074S 1:5000 (Cell Signaling Technologies, Danvers, MA, United States). Blots were developed with chemiluminescence and digitally imaged using a G:Box and GeneSys acquisition software (Syngene, Karnataka, India). Arbitrary gray values of the target bands were obtained using the ImageJ (Version 1.46r; Wayne Rasband, National Institutes of Health, Rockville, MD, United States) gel analysis tool. To more accurately assess protein levels, target bands were normalized to the total amount of protein (derived from Ponceau S staining) measured in their sample lane. Ponceau-normalized lanes were then averaged within each experimental group across 4 experiments (*n* = 4). To calculate the relative change in protein level across the 4 experiments, each averaged gray value was then normalized to the control group for that experiment. All normalized control data are reported as means only, while normalized IRβ data are reported as means ± SEM.

### Data Filtering and Statistical Analysis

To ensure only cells that took up 2-NBDG at a reliably detectable level were analyzed, both neurons and astrocytes were filtered to exclude any cell that did not have a background-subtracted uptake value of 5 or above. After filtering, all cells within a dish were averaged, resulting in an n of 1. We report on 2-NBDG uptake measures of 187 neurons (102 control and 85 IRβ) from 77 dishes (control *n* = 42, IRβ *n* = 35) and 60 astrocytes (32 control and 28 IRβ) from 25 dishes (control *n* = 13, IRβ *n* = 12). For measures of 2-NBDG utilization rates, hippocampal neurons received an additional filter to exclude any neuron that did not have a negative utilization rate (slope) of at least −0.1, as this was calculated to be more than two times steeper than the average background slope of either group (mCherry background slope = 0.1, IRβ background slope = 0.04). This neuronal utilization filter was used to ensure that the reported signal decay was due to biological processes rather than a bleaching effect. We report on 2-NBDG utilization rates derived from 95 neurons (43 control and 52 IRβ) from 46 dishes (control *n* = 21, IRβ *n* = 25) and 60 astrocytes (32 control and 28 IRβ) from 25 dishes (control *n* = 13, IRβ *n* = 12).

**FIGURE 2:**
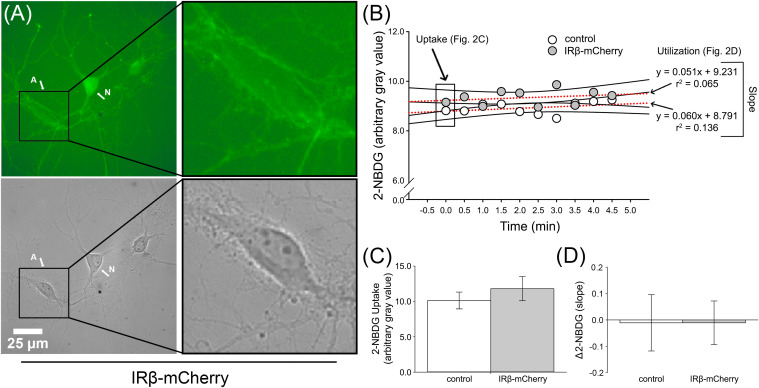
2-NBDG imaging of primary hippocampal astrocytes from dishes with or without IRβ-expressing neurons. **(A)** Representative 2-NBDG fluorescent (top) and phase (bottom) photomicrographs obtained from a single IRβ-expressing hippocampal culture dish. The letter A indicates a distinct astrocyte. The letter N indicates a distinct neuron. Right panels provide greater detail of astrocyte morphology and highlight the visual reduction in 2-NBDG fluorescent signal in this cell compared to the neighboring neuron. **(B)** Data obtained from representative astrocytes from a control or IRβ-treated dish during 2-NBDG imaging. Boxed data at time point 0.0 indicate initial gray value used for 2-NBDG uptake analysis. Dashed red lines represent examples of the linear regressions used for Δ2-NBDG calculation (slope). **(C)** Quantification of background-subtracted 2-NBDG uptake in hippocampal astrocytes from dishes with (*n* = 12 dishes) or without (*n* = 13 dishes) IRβ expression. No significant changes in 2-NBDG uptake values were observed between control and IRβ dishes (Student’s *t*-test, *p* > 0.05). **(D)** Quantification of background-subtracted Δ2-NBDG as indirect measures of 2-NBDG utilization rates in astrocytes. No significant difference in rates of 2-NBDG utilization were noted between control and IRβ dishes (Student’s *t*-test, *p* > 0.05). All data represent means ± SEM.

**FIGURE 3:**
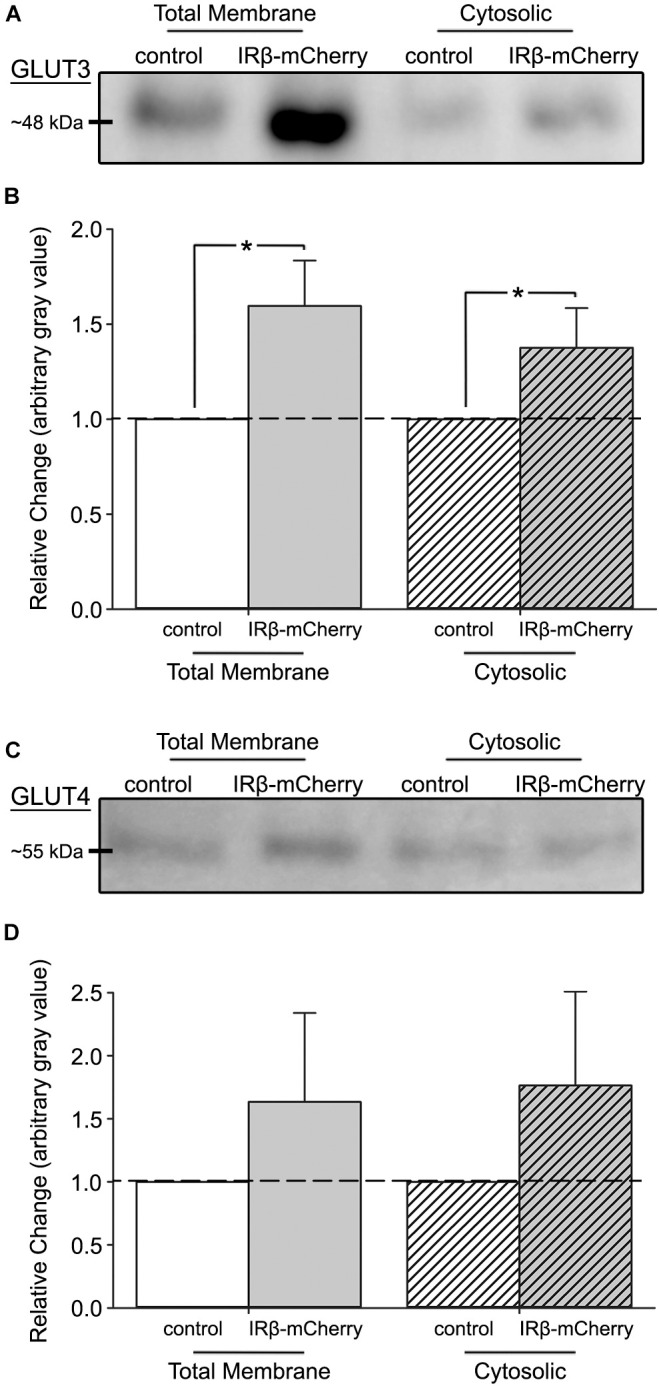
Western immunoblots of fractionated hippocampal cultures with or without expression of IRβ. **(A)** Representative Western immunoblots of subcellular fractions derived from mixed, primary hippocampal cultures (total membrane fraction, left; cytosolic fraction, right) probed for GLUT3. **(B)** Quantification of the relative change in GLUT3 expression between total membrane and cytosolic fractions (*n* = 4 experiments across 5 dams, 8–16 dishes per group). A significant overall effect of virus was detected (2-way ANOVA; *F*_(1,12)_ = 9.36, *p* = 0.010). **(C)** Representative Western immunoblots of subcellular fractions probed for GLUT4. **(D)** Quantification of the relative change in GLUT4 expression between total membrane and cytosolic fractions (*n* = 4 experiments across 5 dams, 8–16 dishes per group). No effect of virus (2-way ANOVA; *F*_(1,12)_ = 1.86, *p* > 0.05) on GLUT4 expression was detected between control and IRβ-expressing cells. All control data represent means. All IRβ data represent means ± SEM. Asterisks (*) indicates significance at *p* < 0.05.

Prior to statistical analysis, all data were first assessed for normality using the D’Agostino-Pearson omnibus test. Data that were distributed normally then underwent a Grubbs analysis to identify and remove any outliers (Figures 1E, 3B,D). Datasets that were not normally distributed were transformed [Y = Log(Y)] and statistical comparison were evaluated on the transformed data (Figures 1C,D, 2C,D). Virus effects on 2-NBDG imaging endpoint measures and ^3^H-glucose uptake were determined using Student’s *t*-tests (unpaired, 2-tailed, equal variance). For GLUT3 and GLUT4 Western immunoblots, virus and subcellular fraction effects on endpoint measures were determined using 2-way ANOVAs with Bonferroni *post hoc* tests. Significance for all comparisons in this study was set at *p* < 0.05.

## Results

### Constitutive Insulin Signaling Conferred by IRβ Increases 2-NBDG Uptake and Utilization Rates in Primary Hippocampal Neurons

To test if chronic, sustained elevations in IR signaling could influence glucose handling, we used 2-NBDG, a glucose analog that indirectly reports on rates of glucose utilization through the loss of fluorescence over time (Natarajan and Srienc, 1999; Pancani et al., 2011). Compared to our previously published 2-NBDG uptake and utilization rates in hippocampal neurons and astrocytes (Pancani et al., 2011), the rates measured here were reduced, likely because these experiments were conducted at room temperature. Nevertheless, it is doubtful this would alter measures unequally in one cell-type compared to another. 2-NBDG was successfully taken up by both neurons and astrocytes. Analysis of initial 2-NBDG images revealed that uptake was significantly elevated in IRβ-expressing neurons (Student’s *t*-test; *p* = 0.046; Figures 1B,C), with some having more than twice the amount of signal compared to controls (Figure 1B). Similarly, IRβ expression was associated with significantly faster rates of 2-NBDG utilization (Student’s *t*-test; *p* = 0.044), as indicated by steeper slopes of signal decay in these neurons (−2.066 vs. −0.548, respectively; Figure 1B,D). Much like measures of 2-NBDG uptake, many of these slopes were more than two-times greater than that of the average control neuron (Figure 1B). Visual observation of the cells during imaging supported our statistical analysis, with neurons from IRβ dishes showing robust and easily distinguishable fluorescence compared to the more subdued signal seen in control neurons (Figure 1A).

Imaging of astrocytes showed no statistically significant difference in 2-NBDG uptake between those from control dishes and those from dishes that received the IRβ plasmid (Student’s *t*-test; *p* > 0.05; Figures 2B,C). Similarly, utilization rates did not differ between the two groups (Student’s *t*-test; *p* > 0.05), and slope averages were relatively flat (control: 0.068, IRβ: 0.035). Visual observation showed a much lower level of fluorescent signal in astrocytes (Figure 2A) compared to neurons (Figure 1A), providing evidence for neuronal-selectivity of the synapsin promoter in the lentiviral constructs. Additionally, the low level of 2-NBDG uptake in astrocytes reported here may reflect their use of alternative energy sources, such as glycogen (reviewed in Brown and Ransom, 2007; DiNuzzo et al., 2010).

### Constitutive IR Signaling Enhances ^3^H-Glucose Uptake in Mixed, Primary Hippocampal Cultures

To corroborate our 2-NBDG imaging data, we performed an additional uptake assay using ^3^H-glucose. Scintillation counts showed a trend for increased ^3^H-glucose signal in IRβ-expressing cells (Student’s *t*-test; *p* = 0.120), which exhibited ∼30% more uptake than control cells (Figure 1E). As differences in cell number between groups could artificially alter scintillation values, we also tested whether the IRβ treatment affected cell viability by comparing total protein load (semi-quantified using Ponceau S staining of Western immunoblots) taken from control and IRβ-treated dishes (*n* = 3 dams, 25 dishes per dam). No significant difference was noted between the two groups (*data not shown*); thus, it is unlikely that variability in cell numbers impacted the ^3^H-glucose uptake values reported here.

### IRβ Expression Elevates GLUT3, but Not GLUT4, Levels in the Total Membrane Subcellular Fraction

To test if elevations in glucose uptake and utilization rates were due to increased GLUT3 or GLUT4 expression, we performed Western immunoblots on total membrane and cytosolic subcellular fractions. Results of GLUT3 immunoblots indicated a significant overall effect of the constitutive receptor (2-way ANOVA; *F*_(1,12)_ = 9.36, *p* = 0.010) (Figures 3A,B), with IRβ-expressing cells having elevated levels of this transporter compared to controls. This was particularly notable in the total membrane fraction, where IRβ correlated with a ∼50% elevation in GLUT3 expression. Surprisingly, no significant differences in GLUT4 expression in either subcellular fraction were detected between control and IRβ-expressing cells (2-way ANOVA; *F*_(1,12)_ = 1.86, *p* > 0.05) (Figures 3C,D).

## Discussion

The current study was conducted to test the hypothesis that sustained, chronic (96 h) IR signaling could influence aspects of glucose handling and/or differentially impact the expression of GLUT3 and GLUT4. We show that constitutive IR activation conferred from IRβ expression is able to significantly increase both glucose uptake and utilization rates, as well as upregulate the total membrane expression of the neuron-specific GLUT3.

### IR Signaling Impacts Glucose Handling in Hippocampal Neurons

Data derived from IRβ-expressing neurons showed that the constitutively active receptor was associated with increased uptake of both 2-NBDG and ^3^H-glucose compared to controls (Figures 1B,C,E). Additionally, we also provide evidence of increased 2-NBDG utilization rates in IRβ-expressing neurons compared to controls (Figures 1B,D). As the rate of glycolysis is directly dependent on the amount of intracellular free glucose and/or previously phosphorylated glucose (glucose-6-phosphate), it is unsurprising that elevations in both measures were detected simultaneously within the same cells. Unlike neurons, astrocytes from IRβ-treated dishes did not show an increase in 2-NBDG metabolism, despite the elevated 2-NBDG uptake and utilization occurring in IRβ-expressing neurons (Figures 2B–D). It has been suggested that astrocytes are metabolically active and may also supply neighboring neurons with lactate produced during anaerobic glucose metabolism (i.e., the astrocyte-neuron lactate shuttle) (reviewed in Magistretti, 2009; Belanger et al., 2011). If neurons primarily relied on lactate supplied by astrocytes, one would expect to see a parallel increase in astrocytic glucose uptake and utilization in response to increased metabolism in IRβ-expressing neurons. However, recent reports have proposed that neurons are capable of independently, and perhaps even preferentially, converting glucose into lactate (Pancani et al., 2011; Lundgaard et al., 2015; Diaz-Garcia et al., 2017). As previously described using a temperature-controlled chamber (Pancani et al., 2011), we show here that even at room temperature, astrocytes appear to have much lower rates of 2-NBDG metabolism compared to neurons. Further, in contrast with prior reports using static (one image) measures of indicator loading (Itoh et al., 2004), the current work provides a dynamic measure of 2-NBDG fluorescence decrease over time. This kinetic profile of lowered 2-NBDG fluorescence at rest, together with reduced metabolism, could be due to differences in glucose uptake, sensitivity to glucose concentrations in the culture medium, as well as differences in the morphology of the cells. However, the similar hexokinase activity in these cell-types, along with prior evidence of increased glucose transport in neurons (Maher et al., 1991; Simpson et al., 2007), corroborate the findings presented here. Lastly, while thinner cell-types (astrocytes) may appear dimmer on an epifluorescence microscope system, this does not necessarily reflect reduced glycolytic rates (Pancani et al., 2011). Overall, the results presented here corroborate prior work from our lab by providing further evidence that neurons are capable of metabolizing 2-NBDG directly and at higher rates compared to astrocytes.

In the periphery, insulin is a key regulator of glucose uptake in adipose and muscle tissue, and it is already known that acute insulin in the central nervous system (CNS) impacts glucose metabolism by altering GLUT4 activity in neurons (Benomar et al., 2006; Piroli et al., 2007; Grillo et al., 2009; Gupta and Dey, 2012; Varshney and Dey, 2016; Reno et al., 2017; Pearson-Leary et al., 2018). Indeed, prior work using 2-(18F)fluoro-2-deoxy-D-glucose (FDG) PET imaging to study brain glucose metabolism in animal models has shown that INI administration can increase glucose uptake following traumatic brain injury (Brabazon et al., 2017) or induced (ICV streptozotocin) IR impairment (Chen et al., 2018), two phenotypes known to present with varying degrees of CNS hypometabolism in the clinic (reviewed in Dietrich et al., 1994; Gross et al., 1996; Soustiel et al., 2005; Baker et al., 2011; Barkhoudarian et al., 2011; Garcia-Panach et al., 2011; Garcia-Casares et al., 2014). It is therefore not surprising that the more sustained IR signaling used here yielded similar results; however, one unexpected and intriguing finding reported in the current study is the observation that constitutive IR signaling selectively increased the expression of GLUT3, but not GLUT4.

### Sustained IR Signaling Regulates the Expression of GLUT3 in the Hippocampus

Results from Western immunoblots highlighted an overall effect of IRβ on GLUT3 expression, with a significant elevation noted in the total membrane fraction (Figures 3A,B). GLUT3 is the primary neuronal GLUT and is distributed within numerous areas of both the human and rodent brain, particularly the cerebral cortex, cerebellum, and hippocampus (reviewed in Nagamatsu et al., 1993; Maher and Simpson, 1994; Vannucci et al., 1997; Choeiri et al., 2002; McEwen and Reagan, 2004). The robust hippocampal expression of GLUT3, along with the evidence of reduced spatial memory in GLUT3-deficient mice (Shin et al., 2018) and the high subcellular localization of GLUT3 to synaptically dense areas such as the neuropil and neuronal processes (reviewed in Mantych et al., 1992; McCall et al., 1994; Maher, 1995; McEwen and Reagan, 2004), suggests this transporter may serve a vital role in learning, memory, and synaptic transmission.

The primary events regulating GLUT3 in the brain are thought to be hypoxia and glucose deprivation (Bruckner et al., 1999; Fladeby et al., 2003; Yu et al., 2008), oxidative stress and variations in fatty-acid availability (Ximenes da Silva et al., 2002; Cidad et al., 2004), brain development and aging (Fattoretti et al., 2001; Rajakumar et al., 2004; Gomez et al., 2010), and neuronal activation and synaptic transmission (Ferreira et al., 2011). While alterations in overall cellular metabolism could have an indirect impact on GLUT3 expression via these regulatory pathways, there is also evidence that insulin and insulin-related processes might be capable of directly influencing this transporter. An early study using cell fractionation techniques showed that both insulin and IGF-I could significantly elevate GLUT3 translocation from the cytosol to the plasma membrane of muscle cells (Bilan et al., 1992), while others have reported that excess thyroid hormones can increase insulin-stimulated recruitment of GLUT3 to the plasma membrane in monocytes (Dimitriadis et al., 2005). Interestingly, recent work has suggested that a similar insulin-mediated modulation may also occur in the brain. In fact, a study in primary hippocampal cultures indicated that *in vitro* administration of insulin significantly increased translocation of GLUT3 vesicles to the plasma membrane, although vesicle fusion of the vesicles and subsequent elevation of neuronal glucose uptake required a KCl membrane depolarization following the initial insulin treatment (Uemura and Greenlee, 2006). While it could be that IR signaling is capable of stimulating GLUT3 translocation through the same process used to mediate GLUT4, other regulatory mechanisms outside of the canonical PI3K pathway should also be considered. For example, recent data has shown that FoxO6, an insulin-sensitive transcription factor abundantly expressed in the hippocampus where it regulates memory and synaptic function (Salih et al., 2012), can also influence aspects of glucose metabolism (reviewed in Lee and Dong, 2017), suggesting a possible role for IR signaling in the transcriptional regulation of glucose-related genes.

Nevertheless, it is important to note that because of methodological limitations when performing subcellular fractionation on primary hippocampal cell cultures (which are grown as non-confluent monolayers) at a single 96 h time-point, the results presented here are not dynamic enough to directly address the impact of IR signaling on GLUT3 translocation to the plasma membrane. Specifically, limited protein yield from the plasma membrane in cultured neurons does not allow us to describe GLUT translocation within these cells. In fact, in all experiments [*n* = 4, each using samples derived from 1 to 2 pregnant dam (12–16 pups per dam)], we were unable to obtain enough protein from the fractionated plasma membrane pellets to reliably perform GLUT3/4 immunoblotting. Therefore, it is necessary to stress that we report only on alterations in GLUT3 expression across the total membrane and cytosolic subcellular fractions at this 96 h time-point, and that these results can not directly address receptor translocation to the plasma membrane.

### Constitutive Activation of the IR Does Not Impact the Expression of GLUT4

Surprisingly, we did not see changes in either the overall protein level or the subcellular localization of GLUT4 following IRβ expression (Figures 3C,D), despite data from several groups showing that canonical IR signaling (e.g., PI3K) in the CNS is indeed capable of modulating this transporter (Grillo et al., 2009) and that this process plays an important role in hippocampally mediated spatial memory (Benomar et al., 2006; Grillo et al., 2009; Pearson-Leary et al., 2018). However, compared to other studies that used acute insulin administration, we induced a chronic activation of the IR signaling pathway, which could perhaps explain the absence of a GLUT4 effect seen here. The brain IR isoform does not appear to be downregulated following sustained signaling (Boyd and Raizada, 1983; Ciaraldi et al., 1985), and neither total IR nor total GLUT4 were reduced in a hyperinsulinemic animal model of peripheral diabetes (obese Zucker rats) (Winocur et al., 2005); thus, constitutive IR signaling may have triggered a compensatory mechanism that prevented a detectable elevation of GLUT4. In fact, recent work from our lab showed that long-term, chronic INI administration (3 months) of insulin aspart did not alter spatial learning and memory on the Morris water maze task in either young or aged Fisher 344 rats (Frazier et al., 2019), whereas more acute, shorter-term exposures (8–11 days) using INI detemir and lispro significantly improved behavioral performance in this same animal model (Maimaiti et al., 2016), adding support to this hypothesis. Further, a study in rats using acute (60 min) ICV insulin showed that while pAKT was elevated throughout this time-frame, GLUT4 expression only transiently increased for the initial 30 min before subsequently returning to pre-insulin levels (Grillo et al., 2009), suggesting that insulin-mediated effects on GLUT4 may be short-lived.

Alternatively, given the important physiological role of microglia in modulating neuronal IR signaling (Holscher, 2019), as well as the presence of IRs and GLUTs 1 and 5 on microglia (Maher et al., 1994; Maher, 1995), it is possible that these cells mediated some of the results presented here. Specifically, neurons expressing the constitutively active IRβ construct may have induced microglial activation, which may have modified the cytokine environment in the culture conditions. However, based on comparisons of protein quantification, we did not note any significant alterations in cell health or density between the two treatment groups. Note that while we did not directly address the potential role of microglia here, the use of mitotic inhibitors may have limited their impact. An emphasis on *in vivo* studies, where microglia activation and function are likely more physiologically relevant, is clearly needed to better characterize this effect.

Finally, it is clear that the use of a molecularly modified, constitutively active IR does not directly parallel the binding of the ligand to its endogenous receptor. While this may underlie the novelty of our results regarding the lack of an effect on GLUT4, the use of this technique allows us to test the impact of long-term, sustained IR signaling and suggests that the approach is viable in neurons. Clearly, additional work investigating the particular pathways and mechanisms involved in insulin’s regulation of brain energy metabolism and its relationship to GLUTs in the CNS is still needed; thus, studies that explore the impact of the modified, constitutively active IRβ receptor *in vivo* are currently on-going in our lab.

## Conclusion

The work presented here demonstrates that: (1) Expression of the constitutively active IRβ receptor significantly elevates 2-NBDG uptake and rates of utilization in cultured hippocampal neurons; (2) Astrocytes may be less metabolically active compared to neighboring neurons; and (3) Chronic IR signaling is associated with increased expression of GLUT3 in neurons. Our results not only support the increasing evidence that IR signaling plays a vital role in brain metabolism, but also suggest a potential novel mechanism (e.g., GLUT3) behind INI’s beneficial impact on learning and memory in the clinic. Additionally, the molecular techniques employed here highlight a new approach to study chronic IR signaling without the need for exogenous ligand delivery.

## Data Availability Statement

The raw data supporting the conclusions of this article will be made available by the authors, without undue reservation.

## Ethics Statement

The animal study was reviewed and approved by the University of Kentucky IACUC Committee.

## Author Contributions

HF and OT contributed to the overall study conception and experimental design. HF and RC performed the experimental manipulations. HF, GP, and MM designed and provided viral constructs necessary for experimental manipulations. LR provided primary antibodies for Western immunoblot manipulations. HF wrote the first draft of the manuscript. RC and LR wrote the sections of the manuscript. OT, AG, KA, R-LL, LR, and RC edited the manuscript revisions. All authors read and approved the submitted manuscript.

## Conflict of Interest

The authors declare that the research was conducted in the absence of any commercial or financial relationships that could be construed as a potential conflict of interest.

## Supplementary Material

FIGURE S1 Western immunoblots of subcellular fractions derived from control hippocampal cultures. **(A)** Top: Representative GAPDH immunoblots of total membrane (total mem.), cytosolic, and plasma membrane (p.m.) subcellular fractions. Bottom: Data were quantified from 2 lanes for the total mem. and cytosolic fractions; due to the low protein yield in the p.m. fraction, only 1 lane was quantified. As expected, GAPDH signal was found in all 3 subcellular fractions. **(B)** Top: Representative calnexin Western immunoblots of the 3 subcellular fractions. Bottom: Data were quantified from 2 lanes for all fractions. As anticipated, calnexin signal was only detected in membrane-containing fractions, suggesting successful separation of the cytosol. All data represent means or means ± SEM derived from a single experiment (*n* = 1).

FIGURE S2 Comparison of Ponceau and Coomassie counterstains for Western immunoblot quantification in the same blot. **(A)** Photomicrographs of the same membrane stained with Ponceau (before probing) and Coomassie (after probing). Similar uniformity across individual lanes is clearly visible using either stain. **(B)** Quantification of GLUT3 and GLUT4 target bands following normalization to either Ponceau or Coomassie yielded similar relationships between the 2 conditions tested (left vs. right). Because this experiment was designed to address the impact of total protein normalization using two different techniques, we did not statistically test for differences between GLUT3 and GLUT4 across conditions tested (*n* = 1); thus, this does reflect the overall means presented in Figure 3 using an *n* of 4. All data represent means ± SEM derived from only 2 lanes in this experiment (*n* = 1).
