# Supplementary figures and images for: Elevating Insulin Signaling Using a Constitutively Active Insulin Receptor Increases Glucose Metabolism and Expression of GLUT3 in Hippocampal Neurons
Source: Front Neurosci. 2020 Jul 7;14:668. doi: 10.3389/fnins.2020.00668 (PMC7358706; doi:10.3389/fnins.2020.00668)

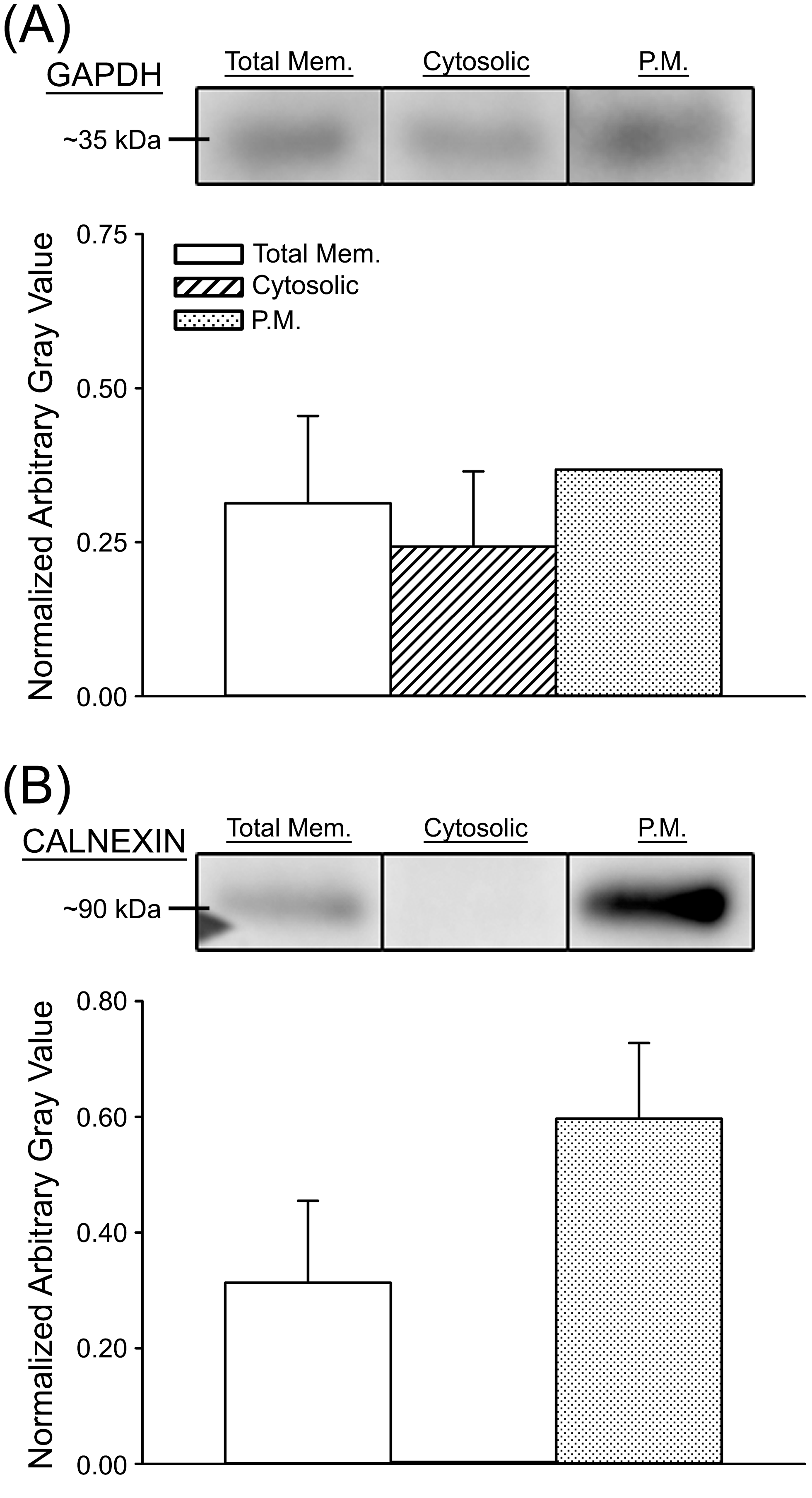

Supplement: FIGURE S1 — Western immunoblots of subcellular fractions derived from control hippocampal cultures. (A) Top: Representative GAPDH immunoblots of total membrane (total mem.), cytosolic, and plasma membrane (p.m.) subcellular fractions. Bottom: Data were quantified from 2 lanes for the total mem. and cytosolic fractions; due to the low protein yield in the p.m. fraction, only 1 lane was quantified. As expected, GAPDH signal was found in all 3 subcellular fractions. (B) Top: Representative calnexin Western immunoblots of the 3 subcellular fractions. Bottom: Data were quantified from 2 lanes for all fractions. As anticipated, calnexin signal was only detected in membrane-containing fractions, suggesting successful separation of the cytosol. All data represent means or means ± SEM derived from a single experiment (n = 1). [file Image_1.TIF]

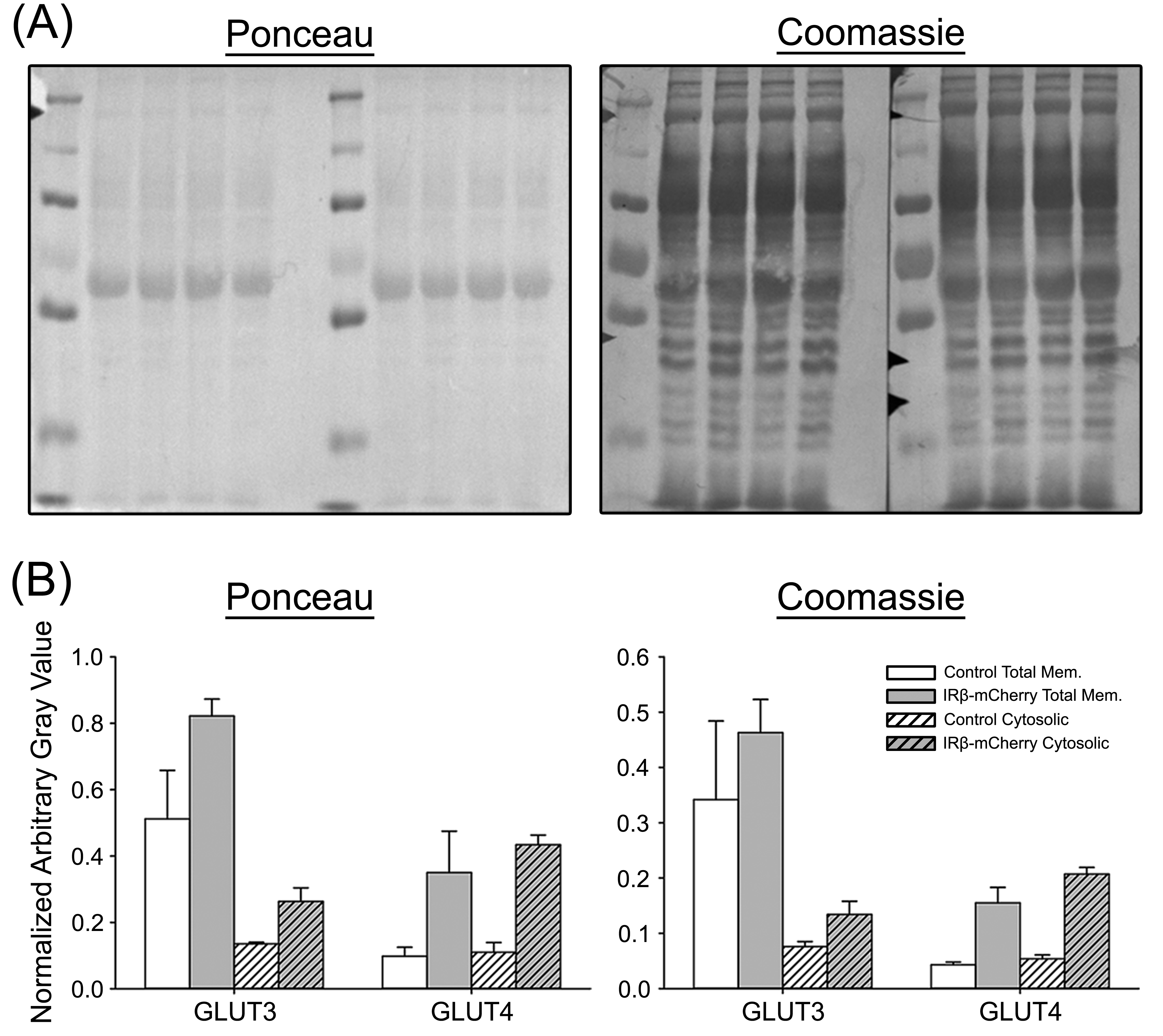

Supplement: FIGURE S2 — Comparison of Ponceau and Coomassie counterstains for Western immunoblot quantification in the same blot. (A) Photomicrographs of the same membrane stained with Ponceau (before probing) and Coomassie (after probing). Similar uniformity across individual lanes is clearly visible using either stain. (B) Quantification of GLUT3 and GLUT4 target bands following normalization to either Ponceau or Coomassie yielded similar relationships between the 2 conditions tested (left vs. right). Because this experiment was designed to address the impact of total protein normalization using two different techniques, we did not statistically test for differences between GLUT3 and GLUT4 across conditions tested (n = 1); thus, this does reflect the overall means presented in Figure 3 using an n of 4. All data represent means ± SEM derived from only 2 lanes in this experiment (n = 1). [file Image_2.TIF]
